# Trends in Physician Work Schedules in Japan: Employed Physician Surveys of the Japan Medical Association in 2009, 2015, and 2021

**DOI:** 10.31662/jmaj.2023-0013

**Published:** 2023-05-10

**Authors:** Tomohiro Ishimaru, Makoto Okawara, Toru Yoshikawa, Michiko Kido, Yoshifumi Nakashima, Anna Nakayasu, Kokuto Kimori, Satoshi Imamura, Kichiro Matsumoto

**Affiliations:** 1 Department of Environmental Epidemiology, Institute of Industrial Ecological Sciences, University of Occupational and Environmental Health, Japan, Kitakyushu, Japan; 2 Research Center for Overwork-Related Disorders, National Institute of Occupational Safety and Health, Japan, Kawasaki, Japan; 3 Department of Obstetrics & Gynecology, Japanese Red Cross Medical Center, Tokyo, Japan; 4 Department of Psychiatry, Mitsui Memorial Hospital, Tokyo, Japan; 5 Executive Boards, Japan Medical Association, Tokyo, Japan; 6 Imamura Clinic, Tokyo, Japan; 7 Japan Medical Association, Tokyo, Japan

**Keywords:** hospitals, Japan, overwork, physicians, sleep

Physician overwork is associated with depressive symptoms and suicidal ideation ^(1), (2)^. The Japan Medical Association (JMA) has been engaged in supporting the health of employed physicians for several years. For example, an email and telephone consultation service has been provided since 2009, workplace improvement workshops have been conducted since 2010, and an analysis and improvement tool for labor management for physicians was published in 2014 ^(3)^. In accordance with the revised Labor Standards Act, physicians will be restricted to 960 hours of overtime per year from April 2024; however, they will be able to work up to an annual overtime limit of 1,860 hours under designated employment conditions ^(4)^. Despite this, the recent work schedules of Japanese physicians remain unclear, and events such as the coronavirus disease 2019 pandemic have dramatically changed the clinical situation ^(5)^. The current study aimed to provide an overview of trends in physician work schedules in Japan.

This study used data from the first (2009), second (2015), and third (2021) nationwide employed physician surveys, which were repeat surveys conducted by the JMA. For each survey, self-administered questionnaires were mailed to 10,000 randomly selected employed physician members of the JMA. We obtained information about age, gender, monthly off-duty days, on-call days, and night shift days, and average sleeping hours on off-duty days from the dataset. Wada et al. previously published the study protocol and questions ^(6)^. In the current study, we excluded respondents aged 60 years or older, and for the analysis, we calculated descriptive statistics for each survey.

We included 3,067, 2,070, and 1,678 respondents in the analysis of the first, second, and third surveys, respectively (Table 1). The percentage of respondents who had eight or more off-duty days per month increased roughly two-fold across the study period (15.8% in 2009, 19.7% in 2015, and 28.5% in 2021). The percentage of respondents with no on-call days increased slightly from 42.3% in 2009 to 48.7% in 2021, whereas the percentage of respondents who had six or more night shift days per month showed a decreasing trend from 12.2% in 2009 to 8.5% in 2021. Sleep hours showed little change, with 9.7% respondents reporting that they slept for less than 5 hours/day in 2009; this proportion was 11.2% in 2015 and 10.0% in 2021.

**Table 1.** Descriptive Statistics of Physician Work Schedules in 2009, 2015, and 2021.

Variable^*^	1^st^ survey (2009) n = 3,067 (%)	2^nd^ survey (2015) n = 2,070 (%)	3^rd^ survey (2021) n = 1,678 (%)
Age, years			
24-39	805 (26.2)	349 (16.9)	496 (29.6)
40-49	1,147 (37.4)	696 (33.6)	432 (25.7)
50-59	1,115 (36.4)	1,025 (49.5)	750 (44.7)
Sex			
Male	2,297 (75.1)	1,542 (77.7)	1,186 (70.8)
Female	762 (24.9)	443 (22.3)	490 (29.2)
Off duty, days/month			
0	282 (9.4)	139 (6.8)	93 (5.6)
1-4	1,293 (43.3)	824 (40.1)	478 (28.8)
5-7	941 (31.5)	686 (33.4)	613 (37.0)
≥8	473 (15.8)	405 (19.7)	473 (28.5)
On-call, days/month			
0	1,281 (42.3)	890 (43.1)	812 (48.7)
1-4	660 (21.8)	457 (22.1)	333 (20.0)
5-7	374 (12.3)	239 (11.6)	182 (10.9)
≥8	716 (23.6)	478 (23.2)	341 (20.4)
Night shift work, days/month			
0	1,126 (37.0)	793 (38.6)	643 (38.4)
1	302 (9.9)	230 (11.2)	162 (9.7)
2-3	682 (22.4)	437 (21.3)	381 (22.8)
4-5	561 (18.4)	368 (17.9)	346 (20.7)
≥6	371 (12.2)	224 (10.9)	142 (8.5)
Sleeping, hours/day			
<5	295 (9.7)	232 (11.2)	167 (10.0)
5-6	1,081 (35.6)	794 (38.4)	555 (33.2)
6-7	1,347 (44.4)	847 (41.0)	770 (46.0)
≥7	310 (10.2)	193 (9.3)	182 (10.9)

^*^ Each variable has missing values and the total numbers do not match.

Our results suggest that physician work schedules in Japan showed some trends toward improvement from 2009 to 2021, although caution must be exercised due to the potential biases in sex, age, and response rate of the survey respondents. Notably, the number of off-duty days for employed physicians has been steadily increasing. Meanwhile, the number of on-call and night shift days slightly changed compared with the number of off-duty days. This gap may indicate that some physicians are engaged in on-call or night shift work instead of taking off-duty time ^(7)^. In addition, because sleeping hours appear to be unchanged, self-study outside work hours should also be considered ^(4)^. Furthermore, lack of sleep is a risk factor for patient-related medical incidents ^(8)^ and may also harm physicians’ health ^(9)^. Further efforts are required to comply with regulations regarding working hours.

## Article Information

### Conflicts of Interest

None

### Sources of Funding

This work was supported by the Japan Medical Association (no grant number).

### Acknowledgement

We thank all members of the Japan Medical Association who participated in or supported this study.

### Author Contributions

TI: conceptualization, investigation, data analysis, and manuscript writing/editing. MO: investigation, data curation, and manuscript reviewing/editing. TY: project administration, investigation, and manuscript reviewing/editing. MK, YN and AN: investigation, and manuscript reviewing/editing. KK, SI, and KM: funding acquisition, supervision, and manuscript reviewing/editing.

### Approval by Institutional Review Board (IRB)

This study was approved by the Human Research Committee at the Institute for Science of Labour (2008-0020), the Ethics Committee of the University of Occupational and Environmental Health, Japan (H27-012), and the Research Ethics Committee of the National Institute of Occupational Safety and Health, Japan (2021N32). Participation was voluntary and anonymous; therefore, the requirement for written informed consent was waived.

### Disclaimer

Kichiro Matsumoto is one of the Honorary Editors of JMA Journal and on the journal’s Editorial Staff. He was not involved in the editorial evaluation or decision to accept this article for publication at all.

## References

[1] Okawara M, Ishimaru T, Yoshikawa T, et al. Working hours, side work, and depressive symptoms in physicians: a nationwide cross-sectional study in Japan. J Occup Health. 2022;64(1):e12377. 36459419 10.1002/1348-9585.12377 PMC9717704

[2] Ishikawa M. Long working hours, depression and suicidality among OB/GYNs in Japan. Occup Med (Lond). 2022;72(3):200-6. 35064662 10.1093/occmed/kqab191

[3] Japan Medical Association. Health support for employed physicians [Internet]. 2022 [cited 2023 Jan 20]; Available from: https://www.med.or.jp/doctor/hospital_based/support/. Japanese.

[4] Wada K, Endo M, Smith DR. New reforms to limit the excessive working hours of Japanese physicians and help prevent karoshi. J Occup Environ Med. 2019;61(6):e304-5. 31166286 10.1097/JOM.0000000000001595

[5] Ishimaru T, Shimizu S, Teshima A, et al. The impact of COVID-19 outbreak on health emergency and disaster in Japan. Sustainability. 2022;14(23):15686.

[6] Wada K, Yoshikawa T, Goto T, et al. National survey of the association of depressive symptoms with the number of off duty and on-call, and sleep hours among physicians working in Japanese hospitals: a cross sectional study. BMC Public Health. 2010;10:127. 20222990 10.1186/1471-2458-10-127 PMC2848631

[7] Harbeck B, Suefke S, Haas CS, et al. No stress after 24-hour on-call shifts? J Occup Health. 2015;57(5):438-47. 26119209 10.1539/joh.14-0276-OA PMC6706192

[8] Kaneita Y, Ohida T. Association of current work and sleep situations with excessive daytime sleepiness and medical incidents among Japanese physicians. J Clin Sleep Med. 2011;7(5):512-22. 22003348 10.5664/JCSM.1322 PMC3190852

[9] Hiyama T, Yoshihara M. New occupational threats to Japanese physicians: karoshi (death due to overwork) and karojisatsu (suicide due to overwork). Occup Environ Med. 2008;65(6):428-9. 10.1136/oem.2007.037473 18487428

